# What role does touch play in active entertainment? A narrative review of tactile feedback in gaming

**DOI:** 10.1177/20416695251336117

**Published:** 2025-05-21

**Authors:** Yang Gao, Charles Spence

**Affiliations:** University of Oxford, Oxford, UK

**Keywords:** active entertainment, gaming, haptics, tactile feedback, multisensory perception

## Abstract

This narrative literature review explores the role of tactile stimulation within interactive gaming and storytelling. Focusing on active entertainment experiences—where the audience/player has some control over what happens, unlike passive media such as film. The review traces tactile/haptic feedback from the 18th century electric-shock amusements to contemporary vibrotactile controllers and interactive cinema. The review also highlights touch's potential to enhance interactivity across active entertainments. Key themes include the role of touch in active versus passive forms of entertainment. In the context of active entertainment, audience participation influences the gameplay/plot, while in passive entertainment, the audience simply observes without being able to alter the outcome. The review also contrasts first- and third-person viewer perspectives and the viewpoint specificity of much tactile/haptic stimulation, particularly concerning these perspectives. Additionally, the review discusses the technical challenges associated with much of the wearable haptic technology that has been introduced to date, and consumer preferences and willingness to pay for haptic devices and enhanced haptic (broadly referring to any kind of tactile) experiences. The review concludes by emphasizing the multifaceted roles of haptic feedback in gameplay, narrative enhancement, and emotional engagement. It also suggests directions for future research to expand the potential of touch within multisensory entertainment.

## How to cite this article

Gao, Y., & Spence, C. (2025). What role does touch play in active entertainment? A narrative review of tactile feedback in gaming. *i–Perception, 16*(0), 1–20. https://doi.org/10.1177/20416695251336117

## Introduction

In 2006, Sony announced that its PlayStation 3 console would no longer include a vibration feature, unlike the PlayStation 1 (1997) and PlayStation 2 (2000), where this feature had been integral to its DualShock controllers. However, a survey conducted by Ipsos-Insight (2006) revealed that 72% of the 1,075 participants believed that rumble/vibration feedback enhanced their game-playing experience “most of the time” in one or more of the following ways: making the game more fun, involving the player more in the action, making the game seem more realistic, and helping to improve the gamer's performance. According to Parisi (2015), it was this feedback, in particular, that led Sony to reinstate the vibration function in its PlayStation 3. This functionality has continued to be a standard feature in subsequent models, including the PlayStation 4 (2013), which also featured DualShock, and further evolved with the DualSense controller on the latest PlayStation 5 (2020), offering finer, more variable, and immersive haptic feedback (Sony Interactive Entertainment, n.d.).

In two recent narrative historical literature reviews (see Spence & Gao, 2024a, 2024b), the use of vibrotactile stimulation to enhance the audience's experience of film and other forms of passive multisensory entertainment were analyzed. Various challenges to the widespread incorporation of digital touch were identified by these reviews, including those of a technological, financial, cognitive, and creative ethical/artistic nature. The reviews highlight that vibration is not widely used in the context of film. Recently, Gao and Spence (2025) further expand on this by exploring the application of touch and haptic feedback in virtual reality (VR) and other interactive entertainment contexts, where their dynamic nature better aligns with the active control offered to users. Similarly, in gaming, vibration has been used more frequently (and thus presumably more successfully). This is in notable contrast to scent, which has rarely been incorporated into commercially successful gaming experiences but has seen rising interest within the film industry—see “scented cinema” (see to Spence, 2020, for a review).

### Active Versus Passive Entertaiment

One of the key challenges when it comes to introducing touch to entertainment is the issue of the audience's passive perspective (Spence & Gao, 2024b; Zhang et al., 2022). While other sensory elements, such as ambient scent, which can serve as an additional cue whose use is less sensitive to the perspective taken, or actively adopted, by the viewer (i.e., the viewer would typically expect ambient scent to affect everyone in a scene more or less equally, no matter who you are/which character you identify with), touch has an inherently interactive nature, which presumably conflicts with passive entertainment. Therefore, touch and touch-related interaction are potentially much more specific to active/passive participant engagement, character, or audience-perspective (i.e., first- vs. third-person). This potential viewpoint specificity can be problematic when the audience is not actively participating, as they may become confused and struggle to follow the plot/character (i.e., if they are presented with a story from a third-person perspective while at the same time experiencing some form of tactile stimulation that appears to refer to a particular character's perspective).

For instance, in a film (or other passive form of entertainment), the feel of a gun being fired should presumably primarily be experienced by the person who pulls the trigger, with no expectation that any of the other characters who may be present in the scene should necessarily feel the tactile sensation (unless perhaps they are the one who has been shot, in which case, the sensation and location of vibrotactile stimulation would likely differ). The location of any simultaneous tactile feedback would presumably help to identify whether the felt vibration relates to the person who has been shot, or the character who is holding the gun: the gun's recoil would presumably be felt in the hand of the shooter, while the impact of being shot would be felt in the body part that was hit, such as the chest or arm. Tactile stimulation is, in other words, often spatially-localized and hence body-site specific (i.e., it makes sense to feel the recoil of a gun in the hand, not on the back). In a game, however, it is mostly going to be clear to the player whether they are the one firing the gun or else have just been shot, thus meaning that there is unlikely to be confusion about character identification in such cases. This clarity is important because confusion in narrative can turn the audience off, reducing their immersion and engagement with the narrative (Bound, 2017). Although, that being said, it is currently unclear how much any such confusion might affect audience immersion and engagement and thus disrupt the flow of the narrative. Once again, this nature contrasts with the case of augmented ambient scent, which essentially has little to no spatial component.^1^

Could active user participation take advantage of touch's more perspective-specific nature to change this dynamic? While it may be challenging to integrate touch into the context of passive entertainment, it can potentially be used effectively in active user contexts such as games, VR, etc., due to its typical perspective-specificity (i.e., where the tactile stimulation is linked to the actions of the participant as the active agent). When users actively participate, their actions align with those of the character's, enhancing identification with the latter, and thus making it easier (more intuitive) to integrate haptic^2^ feedback. Table 1 summarizes the key distinctions between active and passive forms of entertainment, highlighting their distinct attributes, challenges, and commercial success. The dynamic interplay between tactile feedback and user agency is a key driver of engagement, as players feel their actions directly reflected in the gameworld. At the same time, however, note that using haptic stimulation to indicate every single touchpoint the agent experiences could lead to sensory overload or distraction; hence it is essential to selectively identify the key moments where tactile experience should be shared between the user and the character.

**Table 1.** Differences Between Active and Passive Entertainment.

	Active Entertainment (E.g., Gaming, VR, Themed Park)	Passive Entertainment (E.g., Film)
User engagement	High	Low
Perspective specificity	Player-dependent	Creator-dependent
User interactivity	Strong	Weak
Technical complexity	High	Low
Example applications	Game controller (e.g., DualSense, Nintendo Switch), PSVR2, interactive films (e.g., *Possibilia*), haptic wearables.	4D cinemas' motion chairs
Commercial success	Well-accepted: standard in modern controllers	Limited adoption: few successful applications
Challenges	Technical complexity, comfort, individual differences, cost, integration with other modalities, hygiene concerns, limited evidence on immersion	Technical challenges, cognitive issues, cost, creative and ethical concerns, consumer reception

From early scientific experiments through to contemporary multisensory entertainment, the sense of touch has been incorporated in a variety of ways. The idea of introducing touch in active entertainment can be traced all the way back to the 18th century, where the entire entertainment experience sometimes consisted solely of tactile sensation (an electric shock, to be specific), and the arcade electric machines of the late 19th century showcased initial attempts to combine touch in an amusement setting. The use of touch continued well into the late 20th century and continues currently, with the introduction of vibration in video game controllers and its use with wearable technology together with other interactive media, such as VR. Note that the tactile stimulation discussed here is delivered impersonally by technology or digitally, not through one person physically making contact with another, as in the case of immersive theatre or certain forms of theme park entertainment.

### Audience Interaction in Active Entertainment

Audience interaction in entertainment, no matter whether of a personalized or public nature, can be active, passive, or a combination of both, such as when a game starts with a story setup (passive) and then transitions into interactive gameplay (active). In the research discussed in Spence and Gao's (2024a, 2024b) recent reviews, the members of the audience were primarily considered passive recipients of any digital tactile stimulation. However, it is important to clarify that this genre of “active entertainment” refers to any form of media (or activity) where the participants have the power to initiate actions, actively engaging from their own perspective and making decisions that will impact the likely progression, and thus outcome, of the entertainment experience. The key characteristics of this genre emphasize interactivity (requiring the audience to make choices), personal agency/control, involvement, engagement/identification with the story or environment, and sometimes also immersion, thus helping to create a dynamic and participatory form of storytelling. The audience typically becomes part of the story, either as a character or else by identifying with one of the characters. Note how this type of entertainment places the audience at the center of the experience, making them an active agent rather than just a passive observer.

### 1st and 3rd Person Audience Perspective

The core of active and passive entertainment is the manner of user engagement, which is different from first-person and third-person storytelling perspectives/viewpoints in narrative. Active entertainment can also include third-person interactive experiences, whereby the audience/participants influence the actions and decisions of multiple characters or narrative elements. For example, consider how the innovative third-person interactive film “Possibilia” (2014) allowed the members of the audience to impact the evolution of the story by controlling the environmental settings of the two characters (namely a couple going through a breakup, where the user can switch up to 16 scenarios using arrow keys to see the same conversation unfold in varying tones, involving different emotions and actions: calmly smoking and arguing at the doorway, sadly hugging in the laundry room, or angrily fighting in the garden, etc.). In these scenarios, the participant may act as an active overseer or director, impacting the story from a broader, third-person perspective, while still engaging actively and making decisions that help to shape the progression and outcome of the narrative. Note that passive entertainment can also be presented from a first-person perspective, such as the POV (point of view) shots that are sometimes used in films. For example, research (*N* = 183) on Autonomous Sensory Meridian Response (ASMR) videos has shown that first-person perspectives can enhance emotional engagement and identification, particularly in contexts such as food-related ASMR, where the viewer's POV influences their experience (Bailey et al., 2024).

### Contextualizing Active Entertainment

The term “active entertainment” is by no means widely used: Rather, industry professionals and consumers alike are more likely to use, and to be familiar with, terms such as “interactive media,” “virtual reality,” “immersive experiences,” and “interactive storytelling.” The term reflects the trend toward more engaging and personalized forms of entertainment, with the audience's active choices influencing the experience along the way. In this narrative historical literature review (see Ferrari, 2015; Furley & Goldschmied, 2021, on the strengths of this style of review), we primarily explore the historical development of touch's use in active interactive entertainment media, where the audience actively makes decisions that directly influence the progression of the story or the level of interactivity. While providing this historical overview, we also critically evaluate current applications of tactile feedback in gaming and other interactive media and identify key areas for further research and development.

## Early Uses of Touch: On the Emergence of Shocking Sensations

### 1740s: The Electric Kiss, and Other Early Forms of Haptic Entertainment

Understanding the historical context of touch in entertainment may help to provide inspiration for contemporary and future game research involving the tactile modality. Table 2 summarizes key developments in haptic feedback and related technologies. The concept of the tactile augmentation of entertainment can be traced all the way back to the late 1730s during the early exploration of electricity (Parisi, 2013). Here we can find a historical example that represents one of the earliest forms of interactive entertainment that used touch as a central entertainment/game element, specifically through the sensation of electric shock.

**Table 2.** Key Developments in Haptic Feedback and Related Technologies.

Year	Development	Description
1740s	The Electric Kiss	Georg Matthias Bose's early haptic game using electric shocks (Parisi, 2013).
1886	Electric-Shock Arcade Machines	Machines functioning as both entertainment and medical devices (Costa, 1988; Parisi, 2013).
1920s	Gamified Electrotherapy Machines	Devices like Spear the Dragon integrating narrative elements with shock sensations (Parisi, 2013).
1960s	Sensorama	Morton Heilig's multisensory VR experience combining visual, auditory, tactile, and olfactory stimuli (Desnoyers-Stewart et al., 2024).
1900s–2000s	Transition from Shock to Vibration	Games like Vibratory Doctor and art pieces like PainStation replacing shocks with vibrations.
1976	Sega's Fonz	The first arcade game to introduce haptic feedback via vibrating handlebars (Licata, 2021).
1983	TX-1	Arcade game using force feedback technology in the steering wheel (TX-1).
1980s	Earthshaker!	Pinball machine with shaker motor that vibrates to signify an earthquake (Earthshaker).
1997	Nintendo 64's Rumble Pak	Introduction of haptic motors in video game controllers (Buchanan, 2012; Parisi, 2015).
1998	Microsoft's Force Feedback 2 Joystick	Joystick providing rich engagement with force feedback (Orozco et al., 2012).
2006	Sony's PlayStation 3	Introduction of TouchSense technology and inclusion of vibration feedback (Parisi, 2019).
2010s-Present	Wearable Haptic Devices	Development of haptic vests, gloves, and suits like Teslasuit for immersive experiences.
2017	Nintendo HD Rumble	Introduced high temporal resolution and texture representation for nuanced tactile feedback in gaming
2020	Sony's PlayStation 5	Featuring the DualSense controller with advanced haptic feedback and adaptive triggers for immersive gameplay (Doucet, 2024; Sony Interactive Entertainment, n.d.)
2023	PlayStation VR2	Expanded haptic feedback to the head with responsive vibration for enhanced VR immersion (Sony Interactive Entertainment Europe, 2023)

During this period, Georg Matthias Bose, an experimenter working with electricity, created an electric kissing game, known as Venus Electrificata (the electrified Venus). Typically, a woman would stand on an insulated platform while someone behind her generated electricity to charge her body. A brave player (typically a man in those days) would then approach to kiss her on the lips. The electric charge would jump from her lips to his. Sometimes actual physical contact was not even necessary as the charge can jump across a small gap.^3^ Such early forms of “entertainment” experiences can undoubtedly be considered as an example of haptic interaction, whereby the entire experience was built around the delivery of the electric shock (which was felt as a sharp shock). The audience, often composed of upper-class individuals or else those interested in novel scientific demonstrations, apparently found this to be a highly amusing form of entertainment. Indeed, the combination of scientific curiosity and playful interaction was said to have made a great impression on the public at the time (Heilbron, 1999). That said, although the game may have been a spectacular success and a notable attraction (Parisi, 2018), it has largely been forgotten nowadays.

### 1886: Arcade Electricity Machines, Dual Purposes

Since 1886, electric-shock-based arcade machines have functioned both as medical devices and entertainment game machines. During the period when the slogan “electricity is life” was common, the sensation of electric shock was a key element in these games. These early machines functioned as tests of strength and endurance, with participants holding on to metal hand grips while experiencing different levels of electric current. Similar to modern video game controllers (that nowadays often provide some form of vibrotactile feedback—vibrations delivered to simulate physical sensations), these machines provided a sensation of shock. Some, like an 1886 machine, even gave participants a card at the end indicating the maximum voltage that they had managed to endure successfully (Costa, 1988; Parsi, 2013).

Certain electrotherapy machines were presented as both games and therapeutic devices, challenging the players (or patients) to endure and overcome the novel sensation of electricity. They sometimes also promised health benefits, such as treating nervous system disorders and muscular ailments, alongside their entertainment value (Parisi, 2013). Additionally, they were promoted as helping to alleviate urban stress and restore an individual's vitality, thus making electrotherapy more accessible and affordable to the general public.

### 1920s: Gamification and the Narrative of Electrotherapy

After a few years, such electric-shock based “strength and endurance tester” machines gradually began to incorporate additional narrative elements. The shock sensation was used for two primary purposes: (1) to allow players to feel the current and leave with a lingering sensation, making them believe that their money had been well-spent, and (2) to help immerse the player in the world of the experience (Parisi, 2013).

One particularly famous game, Spear the Dragon by the Exhibit Supply Company (1927), had as its primary goal (similar to earlier versions), to see how strong an electric current the player could withstand. However, it was more narrativized: The sensation of the dragon's fire in the game was translated into the current that flowed through the player's skin, charging them with life energy. The shock sensation acted as the haptic representation of the dragon's fire. If the player was able to endure the haptic shock feeling for long enough, then the spear would touch the dragon, signaling the player's victory with a loud ringing bell. According to Parisi, there were also similar games like Le Dirigible (“The Airship”; Jentzsch & Meerz, c. 1929) and Motor-Car Game (with electric shock) (Presser, Moody, Wraith, & Gurr, c. 1924), all of which incorporated “medical electricity within an audiovisual-tactile narrative framework.” According to Parisi (2013), these shock machines enjoyed widespread popularity in Europe and the US up until the 1930s.

### 1900s–2000s: The Transition from Shock to Vibration

Due to the discomfort caused by electric shocks and the increasing fear that they might harm the user's/player's body, shock sensations were gradually replaced by vibration. With the “golden age” of electrotherapy past (from 1880 to 1920, according to Peña, 2003, p. 99, from Parisi, 2013) and people's fear of electricity rising, the production of electric shock games was eventually discontinued by manufacturers. Consequently, this led to the emergence of two new types of games (Parisi, 2013): Vibration-based games and those games where a mild shock element was reintroduced.

Initially, when game and medical machines transitioned from shock to vibration, they continued to mimic the shock machines that had preceded them. Around 1904, under the slogan “electricity is life,” games promoting “vibration is the law of life” started to emerge, such as the three-armed Vibratory Doctor by the Watling Manufacturing Company. Such games replaced shock with vibration, while at the same time still claiming to offer similar benefits to the user/player. Despite being vibration machines, they initially imitated the shock sensation in gameplay. The Vibratory Doctor represented a transformation in the relationship between electricity and touch, shifting from direct electrical skin contact to motor-generated vibrations.

In the early 2000s, some game art pieces reintroduced the sensation of shock. For instance, games such as PainStation and Tekken Torture appeared, where shocks now served as a form of punishment rather than as periodic elements of gameplay. The shocks inflicted pain and controlled the player's ability to move, but at the same time they ensured the player's safety. In this context, the painful shock was only suggestive of bodily harm without actually necessarily causing it (Gallace & Spence, 2014).

## Touch in Video Games: Vibrotactile Sensations

### 1976: Introduction of Haptic Feedback in Arcade Games

The introduction of vibratory feedback in arcade games can be seen as a continuation of earlier attempts at multisensory stimulation. Morton Heilig's Sensorama in 1962, which was designed to create a fully immersive VR experience using visual, auditory, tactile, and olfactory stimuli, was an early attempt along just such lines (Heilig, 1962). Building on these earlier innovations, Sega's Fonz, introduced in 1976, was the first arcade game to introduce haptic feedback (Licata, 2021). This motorcycle racing game made the player feel the vibration from the handlebars whenever they collided with other drivers or happened to veer off the road (PatmanQC - History of arcade game documentaries, 2019). In the context of the present article, it is worth noting how the introduction of haptic feedback in this game achieved significant market success in the US and Japan. The Fonz was introduced at the MOA Expo in Chicago, where, according to Sega, the response was “unanimous and enthusiastic” (Cashbox, 1976), and it became one of the top 10 bestselling games in Japan at the time (Kirginas, 2022). Such success inspired further developments in arcade games, such as TX-1 in 1983, where force feedback technology was used to make the steering wheel vibrate, thus enhancing the realism of gameplay. Both TX-1 and its sequel were very popular in Japan (TX-1). Besides racing games, pinball machines like Earthshaker (1989),^4^ the first machine with a shaker motor that was themed around California and Nevada splitting apart, with the gap becoming a rail for the pinball. The game, which was released in 1989, also incorporated haptic feedback to simulate “earthquake-like” vibrations and rumbles, with the marketing slogan “It's a moving experience!” (Earthshaker), providing what was claimed to be an immersive and fun experience for players (TNT Amusements Inc, 2013).

Over the years, some arcade games have included an element of physical interaction/touch. Indeed, pinball arcade games once included a tilt warning if the player hit the sides of the machine too hard. This stereotypical form of physical interaction has evolved in certain modern digital pinball machines, where the controller vibrates to mimic the feeling of the cabinet shaking when the tilt feature is activated (Pinball Cabinet, 2023). Unlike many other games, playing an arcade pinball machine therefore involved a degree of physical interaction with the machine itself. Skilled players often deliberately nudge the machine physically in order to help guide the ball's trajectory or bang the side of the pinball machine to get the balls onto the flippers, all while skillfully avoiding the tilt alarm that would freeze the game if the player's nudge was too vigorous.

### 1997–2000s: Vibration Motors Incorporated into Video Game Controllers

Haptic motors were first introduced commercially in video game controllers in 1997, with Nintendo 64's Rumble Pak add-on component (Buchanan, 2012; Parisi, 2015). This enabled players to directly feel the events and movements in the game, increasing immersion, interaction, and the overall gaming experience. In 1998, Microsoft also released its Force Feedback 2 Joystick, which offered rich engagement and, as the name suggests, force feedback (Orozco et al., 2012). Since then, modern controllers have adopted the same design concept, featuring offset motors generating vibration from close to the player's palms (Söderström et al., 2022). The motors are used to deliver a small range of vibrotactile feedback corresponding to different in-game events and scenarios. However, it is important to note that in terms of the location of any vibrotactile feedback, tactile sensations were mostly confined to the player's hands which hold the controller.

Parisi (2009) describes the ‘*tactile semiotics*’ of rumble “are understood in connection with feedback from other modalities, contributing to the modelling and differentiation of game objects and aspects of the Gameworld – for example, helping to tell apart one weapon as distinct from the next, due to different vibrational patterns” (Parisi, 2009, 120; quoted in Willumsen & Jaćević, 2019, p. 2). This application of different haptic feedback technology, where different vibration patterns and intensities are applied to enhance the player's ability to distinguish between in-game actions or objects, extends to various types of games. For instance, in a first-person shooting game, the controller might deliver different patterns and intensities of vibration in order to help discriminate between the various weapons. In a fighting game, the controller is used to generate various patterns of vibration in order to represent the intensity of the impact on the player (Parisi, 2015). It is important to note that while touch and vibration can convey specific information in a game, such as serving as game cues, their full meaning is often enhanced by (or can only be interpreted in terms of) the visual or auditory context in which they are experienced. The vibration of the controller in-and-of itself does not have much of a meaning – it is just a vibration. As such, its meaning typically needs to be clarified by reference to whatever is happening visually or auditorily. Moreover, the effectiveness of this haptic feedback also depends on how quickly the participant can learn and associate the tactile sensations with the corresponding audiovisual context. Despite its continued presence in digital games and game controllers, investigations of rumble, as well as those of general haptic feedback in relation to digital games, are made difficult due to what Lipkin perceives as haptics' lack of “the complex system of signification found in discourses on visuals and sounds” (Lipkin, 2013, p. 36). Further research and evidence are needed to determine the potential impact of this feedback on gaming immersion.

Parisi (2015) pointed out that despite the vibration function being considered “last-gen” by Sony and Microsoft, both companies still chose to include it in their redesigned game controllers, with Microsoft even adding a vibration feedback motor called “Rumble Triggers” to the device's triggers to further enhance the experience. However, the adoption of haptic technologies in game controllers has not occurred without its challenges. Legal issues have undoubtedly played an important role in the incorporation (or not) of haptic technologies in game controllers. In particular, Immersion Corporation filed lawsuits against both Sony and Microsoft for patent infringement on their haptic feedback technology. Due to the ongoing legal battle at the time, Sony initially omitted vibration feedback from the PlayStation 3's SIXAXIS controller. Eventually, however, due to the suggested importance of vibration for the gaming experience, as highlighted by the Ipsos-Insight (2006) survey mentioned in the Introduction, Sony settled with Immersion and reinstated the vibration feature in its DualShock 3 controller (Parisi, 2015). Microsoft also settled with Immersion in 2003 with a secured licensing agreement that allowed them to continue using the technology.

### Vibration Feedback in Contemporary Gaming

The gaming industry has long wanted to augment entertainment experiences using vibrotactile/haptic stimulation to enhance immersion in video games. In 2006, Immersion Corporation introduced its “TouchSense” technology in order to enhance the realism of video games by synchronizing haptic feedback with the relevant on-screen audiovisual events (Parisi, 2019). This technology allowed for a more immersive gaming experience by providing tactile sensations that corresponded to various in-game visual and auditory stimuli. For example, the Nintendo Switch used this technology to improve game experiences through its Joy-Con controllers (Seedhouse, 2017). Meanwhile, Immersion Corporation published a report on best practices for vibrotactile feedback, thus providing valuable design guidelines for game developers and emphasizing the desirability and importance of vibration feedback (Immersion Corporation, 2010).^5^ In 2017's Nintendo Switch, it also incorporated HD Rumble, a technology that uses temporal resolution and texture representation to enhance the user's experience. For example, in the game *Super Mario Odyssey*, HD Rumble provides rapid tactile feedback, allowing players to locate hidden Power Moons (as reward) to progress in the game. Similarly, temporal resolution and texture representation are also used in PS5's DualSense controller. For instance, as demonstrated in *Astro's Playroom*, players can feel surface details like grass, sand, metal, and water through the controller's haptic feedback. Additionally, the adaptive triggers provide dynamic sensations, such as the thruster ratting against the player's finger during gameplay (Doucet, 2024)^6^.

When thinking about adding tactile feedback, one should also consider what kind of game it is going to be integrated into, just as in the case of film. For example, “most often, shooter games benefit from rumble packs because it puts the feel of a real weapon directly in your hands” (Immersion Corporation, 2010). For instance, the PS5 game “Call of Duty: Modern Warfare II” includes force feedback, allowing players to mimic shooting an arrow when pressing the button on the controller. This feature simulates the force feedback of the arrow, including the tension of a bowstring when performing archery actions. The adaptive triggers can mimic the force required to pull back the bowstring, making the action feel more realistic.

While vibration feedback is the most commonly recognized form of haptic interaction in contemporary gaming, researchers also explored more advanced haptic rendering algorithms that simulate detailed physical interactions. Andrews et al. (2006) present the game “HaptiCast,” which serves as an experimental framework for assessing haptic effects in 3D games. In HaptiCast, players act like a wizard and use haptically-enabled wands to interact with the game world. Players can feel the physical properties of in-game objects and the game integrates haptic rendering algorithms that allow players to feel textures, weights, and force, thereby enhancing the interactive experience, which showcases how haptic feedback in gaming can extend beyond simple vibrations to include a broader range of tactile sensations and embodied interactions, where the way players move their hands/bodies also plays a important role in the overall gaming experience.

When discussing the location of vibrotactile feedback, it is important to note that most tactile feedback has been focused on the hands. Quintin Morris from Microsoft noted that the feedback is likely to be most effective on the fingertips, the most sensitive part of the hands, and one of the most sensitive parts of skin surface (Gallace & Spence, 2014; Parisi, 2015; Weinstein, 1968; Wilska, 1954). Some games, such as Ring Fit Adventure on the Nintendo Switch, allow the user to attach the controller to their leg in order to feel vibrations while playing. Other games, meanwhile, feature wearable devices that sometimes provide vibrations on the back, or on participant's gluteal area or lower body using the ButtKicker Gamer (Lovreglio et al., 2018). VR devices, such as PlayStation VR2 (PSVR2) have expanded vibrotactile feedback to the head. For instance, in *Horizon Call of the Mountain*, the players can experience “subtle, responsive headset vibrations at key moments,” which contribute to a more immersive experience (Sony Interactive Entertainment Europe, 2023). However, the effects of applying tactile feedback to different body parts on improving the entertainment experience, user engagement, and immersion are still largely unknown (see Gallace & Spence, 2014 for a review).

The integration of advanced haptic feedback in modern multiplayer online role-playing games (MMORPGs), could potentially also significantly enhance the gaming experience. For instance, devices such as haptic gloves, vests, and other wearable devices could offer a more realistic and engaging gaming experience in MMORPGs, where multiple players interact in a shared virtual world. There is, though, a questionmark about tactile stimulation when its elicitation is triggered by other players, rather than carefully programmed by the developers. One could also imagine multiple players wearing controllers on their legs and racing together in online games similar to Ring Fit Adventure. Smaller-scale two-player digital games such as “It takes Two,” could also be enhanced by the incorporation of haptic feedback. In particular, when one player dies, both players receive vibration feedback via their controllers. However, it is uncertain how much people would be willing to pay to have such additional tactile feedback.

### Interim Summary

Vibrotactile feedback has become an integral component in modern gaming, enhancing not only intense gameplay scenarios but also more routine and relaxing activities. For example, in the game Animal Crossing: New Horizons (Takeshita & Miyake, 2020) on the Nintendo Switch, the Joy-Con controllers vibrate whenever the player's digital avatar is fishing or engaging in DIY crafting, aiming to create a more engaging and immersive island experience. The roles of haptics in games are multifaceted: in particular, haptics can serve as game cues, serving to enhance the player's awareness and interaction with in-game events. Furthermore, haptics can be integrated with various wearable devices to enhance the gaming experience. For instance, wearable devices such as haptic gloves, suits, and backpacks can be used to deliver localized haptic feedback to different parts of the body, potentially contributing to a more immersive experience. Although less common, haptics can also function as an element in the narrative, enriching the storytelling aspect of games by adding a tactile dimension, akin to how one could compose tactile music (Gunther, 2002). Through these various applications, haptic feedback may not only enhance the sensory experience of gaming but also potentially strengthen the emotional and psychological engagement of players. Additionally, the vibration of the controller on its own does not carry much meaning. Instead, its meaning typically needs to be interpreted in conjunction with the accompanying visual or auditory cues.

## The Function of Touch in Active Entertainment

### Game Cues

Haptic feedback can serve as a cue in the context of gaming (de Menezes & Lamar, 2023), providing players with information that they may not otherwise notice. For example, in the Nintendo Just Dance (Paris et al., 2009–2025) game, players hold the joystick in their hands while performing dance movements on the screen. However, the scoring bar on the side might provide too much information for the players to focus on while simultaneously trying to concentrate on each of the dance moves that they need to execute. To address this, *Just Dance* combines vibrational feedback from the joystick with auditory cues, such as celebratory sounds, and visual elements, like flashing animations on the screen, to indicate when players perform a movement correctly and achieve a high score. The multisensory feedback system allows players to understand their performance through multiple input channels, rather than relying solely on one sensory modality, such as visual information. By integrating touch, sound, and visual cues, the game helps reduce cognitive load (Gallace & Spence, 2014; cf. Ngo et al., 2012) and allows players to remain more focused on their movements and immersed in the game.

Haptic feedback may, of course, also serve as game cues to assist those players who are hard of hearing or deaf (de Menezes & Lamar, 2023). For instance, *The Laste of Us Part I* introduces a feature where spoken dialogue is converted into vibrations on the PS5 DualSense controller, allowing deaf players to “feel” the delivery of lines of dialogue (McAllister, 2022). Haptic cues are also beneficial in public settings where the sound of gameplay may not be convenient or socially acceptable if it is too loud, and so disturbs others (Tao et al., 2021). Note here that people often turn off the sound (or, in the case of children, are told to do so by their parents) in order to avoid disturbing others nearby when playing in public (Ekman, 2007). This is one of the situations in which haptic cues come into their own, transmitting cues to give players notifications and increase the immersion of the game.

### Touch With Wearable Devices

Researchers have begun to explore innovative haptic feedback devices beyond just controllers, such as wearable devices, to provide embodied experiences. For example, Palan (2020) developed a vibrotactile vest that was designed to mimic the heat and impact of being hit by a bullet. The initial goal is that this may help to increase the immersion of shooting games. LifeTact by Pescara et al. (2017) uses smartwatch vibrations to remind the user of their remaining life in the game through variations in the frequency and presence of a tactile heartbeat, potentially enhancing the gaming experience.

Additionally, researchers have also explored the use of everyday objects to help deliver haptic feedback in a more bespoke, if less ubiquitous, manner (Lee, 2011), thus potentially making the multisensory experience more familiar, accessible, and immersive. These advances could contribute to other broader entertainment contexts in the future, such as Augmented Reality (AR) applications, theme park experiences, as well as others (Spence, 2022b, 2022c).

### Narrative Elements

Haptic feedback can potentially enrich storytelling by adding a tactile dimension to the narrative and symbolizing in-game events, thereby enhancing the player's connection to the story. It can also serve as a core element in driving a game's narrative (like the Electrified Venus and Spear the Dragon mentioned earlier). For example, research has shown that in the game “Glow the Buzz,” incorporating haptic feedback as the primary means of interaction can make the game more engaging and immersive (Jeong et al., 2023). The game uses three types of haptic stimulations—rhythm, texture, and direction—each designed to provide unique means of tactile feedback. Iterative game testing conducted by Jeong et al. (2023) with a group of 10 participants demonstrated that players were clearly able to distinguish between different tactile stimuli and rated their experience highly in terms of engagement and on immersion metrics. Specifically, the study reported scores on the System Usability Scale for presence in VR (average score of 5.4 out of 7) and on the Interest/Enjoyment subscale of the Intrinsic Motivation Inventory (average score of 5.8 out of 7), indicating a positive reception to the haptic feedback as the primary method of interaction. The study also indicated that players enjoyed the novelty of using touch as the primary means of interaction, which added a unique layer of physicality to the gameplay that more traditional visual and auditory feedback could not achieve. (Though of course, the novelty angle is likely to be rapidly lost the more commonly vibration is used.) The small sample size (*N* = 10) and the novelty effect of the haptic interface may limit the generalizability of these findings.

The ability to distinguish between different tactile stimuli can vary (sometimes quite dramatically) depending on the area of the skin surface being stimulated (Gallace & Spence, 2014; Weinstein, 1968). People's ability to resolve different tactile stimuli is generally limited, often only able to distinguish between low, medium, and high-intensity stimuli. So the design of tactile interactions must take these perceptual constraints into account. People also differ in terms of how easy they find it to direct their tactile attention to different regions of the body surface too (Pritchett et al., 2011; Spence & Gallace, 2007). Based on all the various functions that touch could potentially play in the context of entertainment, Table 3 provides an overview of the various roles that touch can play in these settings.

**Table 3.** Examples and Impact of Tactile Interactions in Games and Entertainment.

Function	Example	Types of Tactile Stimulation	Effects
Gameplay elements	The Electric Kiss (1740s) (Parisi, 2013)	Shock	Provided amusement and curiosity, added suspense and excitement.
Narrative elements	Glow the Buzz (2023) (Jeong et al., 2023)	Vibration	Enhanced engagement and immersion, added unique physicality to gameplay.
Game cues	Nintendo Just Dance (2009–2025)	Vibration	Assisted player focus, enhanced immersion, aided accessibility for hard-of-hearing.
Wearable devices	Vibration Vest (Palan, 2020), LifeTact (2017) (Pescara et al., 2017)	Vibration	Increased immersion, provided life reminders, potential discomfort, and cost concerns.
Health and therapy	Strength and Endurance Testers (1886) (Parisi, 2013)	Shock	Immersed players in narrative, tested endurance, provided a sense of achievement.

## Current Challenges in Haptic Technology in Games

While technologies such as the Woojer Vest Edge have made advancements in providing low-latency haptic feedback for gaming (Woojer, n.d.), fully achieving accurate and spatiotemporally synchronized haptic feedback across multiple body sites remains a significant technical challenge, highlighting the need for further experimentation in order to determine the most immersive, comfortable, easy-to-use, and overall satisfying use of haptics on different body parts, in line with principles of user experience. At the same time, many of the wearable functions discussed remain in experimental stages and are all possible but not yet available for commercial use due to high production costs, limited scalability, lack of generalization among diverse users with individual differences in sensitivity, preferences, and body characteristics, and usability challenges. Moreover, the discomfort and delays associated with some devices further hinder their adoption in real-world gaming scenarios. Additionally, future research should probably explore how individual sensitivity to vibration varies, as some users may perceive the same tactile stimulation as too weak or too strong (Cholewiak, 2019). It should, however, be noted that wearing haptic devices can sometimes be uncomfortable, as reported by participants in the studies by Jeong et al. (2023). The weight of certain of these devices has also been reported to contribute to user discomfort (Han et al., 2018).

Therefore, haptic feedback should be carefully balanced to avoid any possibility of overwhelming or confusing players (e.g., when trying to interpret the meaning of ambiguous feedback). Additionally, it is important to consider that haptic devices like vests or seats are often worn over people's everyday clothing, which can further contribute to individual differences in perceived vibration intensity (cf. Ho & Spence, 2008; Van der Burg et al., 2009).

Moreover, if haptic feedback does not complement or integrate well with visual and auditory cues, players may also find it distracting (Kirginas, 2022). Thus, ensuring accurate spatiotemporal synchronization of haptic feedback with other sensory inputs is likely to be crucial to user/gamer acceptance. At the same time, however, further research is needed in order to determine how sensitive people actually are to haptic asynchrony in the context of storytelling/gaming, as even slight mismatches can potentially cause confusion and at the same time reduce the sense of immersion and realism, similar to mismatched audiovisual cues in the context of film (Gallace & Spence, 2014; Spence, 2014).

While vibration is a standard feature in game controllers, the willingness of consumers to pay for this feature is not clear. For instance, the Nintendo Switch Lite, which lacks a vibration function and is priced below the standard Nintendo Switch, hints at a possible trade-off between cost and sensory functionality. In the 2006 Ipsos-Insight report, only 46% stated they might still purchase a PS3 or be even more likely to purchase it if it did not include the vibration function. Additionally, 58% of the respondents expressed disappointment with the lack of vibration feedback in PlayStation 3 controllers (Ipsos-Insight, 2006). Research by King et al. (2024) also showed that adding a sensory signal to products can improve overall evaluations and repurchase likelihood. It would be interesting to know how responses would change if the price implications of tactile/haptic augmentation were to be made explicit to the consumer. Similarly, the willingness to pay for other forms of enhanced haptic experience, such as pressure feedback, temperature feedback, haptic suits, and more (even assuming that they were commercially available), also currently remains unclear. Understanding the consumer's preference for, and the value placed on, haptic feedback is presumably going to be crucial for pricing and developing future touch-enabled gaming devices.

Potential hygiene concerns associated with tactile stimulators that touch the skin should be considered, especially in shared environments like video game arcades, where multiple users handle the same devices. In a post-COVID world, implementing cleanliness rules and regular sanitation could help ensure the hygienic use of tactile technology in these public entertainment contexts (Makela et al., 2021), while at home where the same people repeatedly use controllers, the risk may be lower. It also provides one reason to prefer non-direct touch tactile stimulation devices, such as provided by Ultrahaptics (e.g., and as incorporated at Tate Sensorium, where more than two thousand viewers experienced the same tactile stimulation (Pursey & Lomas, 2018), or gestural interfaces like Leap Motion, used in Rober Yang's Hurt Me Plenty (Pozo, 2018).

Researchers have tried to incorporate haptic feedback in order to make games more immersive and engaging, it turns out that there is still surprisingly little experimental evidence to support such claims (i.e., of increased immersion). Consistent research evidence demonstrating the benefits of tactile feedback for immersion remains limited. While studies such as that of Jeong et al. (2023) show promising results, these findings are often constrained by small sample sizes, potential novelty effects, and the controlled nature of laboratory environments. Moreover, consumer expectations and preferences for tactile feedback have likely evolved since earlier studies, such as the Ipsos-Insight survey (2006), which is now nearly two decades old. These limitations highlights the urgent need for further studies to empirically investigate the impact of haptic feedback on player immersion and engagement, setting the stage for our own future research in examining these effects in various real-world gaming contexts.

It is also important to note a potential limitation of haptic technology: namely, the phenomenon of tactile inattentional blindness, whereby an individual may fail to notice tactile stimuli when their visual (and/or perhaps auditory) attention is occupied elsewhere (see Gallace & Spence, 2014; Murphy & Dalton, 2016). This might influence the effectiveness of tactile feedback if the player happens to be too focused on the visual (or auditory) aspect of the game. On the other hand, however, there is also a literature from experimental psychology showing how the presentation of occasional sudden-onset tactile stimulation can also help to draw a person's attention to whatever events happen to be occurring simultaneously in a different modality (e.g., Spence & Ngo, 2012; Van der Burg et al., 2009).

Despite these challenges, the widespread belief amongst many of those working in this area is that technology-enabled haptic sensations in gaming can significantly enhance the overall gaming experience. Yet, the question still remains: Are consumers willing to pay for such enhanced multisensory experiences? Addressing these limitations, such as relating to cost, complexity, individual differences in tactile sensitivity, and user comfort, will hopefully make the multisensory game experience more immersive.

## Conclusions and Future Research

This narrative historical review highlights the surprisingly long history of touch in gaming and entertainment, dating back at least as far as the 18th century. It remains a popular feature in contemporary gaming controllers as well as other forms of active entertainment. Despite the popularity of vibration feedback in the context of gaming, its application in film has not seen similar uptake, nor success (see Spence & Gao, 2024a, 2024b). This fundamental difference raises some intriguing questions about the varying roles that touch may play in the context of active versus passive entertainment, as well as the relevance of touch in first-person versus third-person viewer perspectives. Touch appears to be more relevant/appropriate in the context of active (rather than passive) forms of entertainment, such as video games, VR, or in film contexts where the story is more about “me” (the audience), for example, experiencing a POV shot or global event like feeling the chair rumble during an earthquake movie. Here, participants directly experience sensations as a result of actively engaging and identifying with the character/settings. In active entertainment, players directly interact with the environment and make decisions that affect the narrative, enhancing their immersion and connection to the virtual world. In contrast, visual and auditory elements are relevant and critical to both first-person and third-person perspectives. By comparison, olfactory stimulation is seemingly appropriate across a wider range of contexts (see Spence, 2020).

While touch plays an important role in enhancing experiences in active entertainment, its implementation need further research and investigation. Wearable haptic devices in gaming and other interactive media settings face challenges such as possible delays in the response to user operations and potential user discomfort. The effectiveness of these devices in terms of enhancing user immersion is currently not well-established. Furthermore, what constitutes the optimal placement of vibrotactile effects/stimuli on the body (assuming that the technological means of delivery were not an issue) is still a subject of active investigation. Simplifying haptic effects and integrating them seamlessly with visual and auditory feedback will likely also help to improve the user experience (Kirginas, 2022). In 2021, David Birnbaum, the Senior Director of Immersion Corporation, stated that the future of gaming will feature multi-channel haptics, similar to multi-channel audio, where haptic effects can be applied on and around the body (Birnbaum, 2021). For example, games such as Nintendo's Just Dance and Ring Fit Adventure apply vibration feedback to different parts of the body.

These technical challenges and possibilities raise broader question about how touch is experienced and used differently across various forms of entertainment. As highlighted by the present review, the success of vibrotactile feedback in gaming, as compared to its limited uptake in cinema, may be attributable to the active engagement of players in games versus the passive experience of film audiences. Additionally, in terms of the location of the tactile feedback, players' hands are naturally more sensitive and provide an easy way to incorporate touch, especially since they are already in contact with the game controller. Game controllers have also become branded elements of the gaming experience. In contrast, in cinema, the primary point of physical contact is the seat, and the backside is simply not as sensitive as other parts of the body surface. This raises the question of whether interactive films, which involve active participation from viewers using devices like a mouse, keyboard, or controller, could benefit from some form of haptic feedback. Unlike traditional films, where the audience remains passive, interactive films could use the same sensitive areas of the hands that are used in gaming to enhance the viewer's experience. Comparing the impact of vibration in interactive films and traditional films based on the same story could offer insights into the potential of haptics in enhancing narrative experiences.

In the context of gaming, tactile stimulation can potentially serve as a narrative element, as seen in contemporary games like “Glow the Buzz” (Jeong et al., 2023). For touch to effectively enhance the narrative, it may need to fulfil specific conditions, such as providing unique sensory experiences that cannot be conveyed visually or auditorily (e.g., the use of mid-air haptics in the VR experience AFFECTED: The Visit by Fallen Planet using a Ouija board Blenkinsopp (2019)). The role of sensory augmentation in merely pleonastically repeating on-screen actions (cf. Banes, 2001; Spence, 2021), which is often ineffective in films, might have different implications in interactive and immersive gaming contexts.

Overall, the integration of touch in active entertainment potentially plays several roles: being part of the gameplay/narrative, serving as game cues, being used with wearable devices, and potentially even having health and therapeutic functions. These roles enhance the audience/participant's immersion, engagement, and emotional connection. While challenges undoubtedly do still remain, such as the appropriate provision and ownership of the technology, consumers typically purchase the necessary equipment in gaming. In the context of cinema and other passive forms of entertainment, the responsibility often lies with the providers to incorporate and maintain the appropriate technology, such as upgrading chairs for additional sensory experience, or providing 3D glasses. Therefore, further research is needed to expand the potential of touch in active entertainment and develop guidelines for its use in other multisensory entertainment contexts.

Additionally, scenarios can be imagined in which various tactile elements are added to film or gaming experiences independently of the creator, in a similar way to how Ultrahaptics was incorporated into art viewing at the Tate Sensorium a decade ago, by adding other sensory tracks such as haptics and sound, created by people other than the original artists (Pursey & Lomas, 2018). However, it should be recognized how this can potentially raise ethical/creative questions about altering an artist's or creator's original work without their consent (which may be more of a concern in the context of the art gallery than it is in gaming, where some game controllers even allow players to program their own haptic feedback). In gaming, incorporating touch could also enhance game performance by helping to reduce visual overload (cf. Spence & Ho, 2008). However, if that were the case, one has to ask where the evidence supporting such a beneficial effect is. This would certainly seem like an important and appropriate area for future research.

Exploring diverse types of tactile stimulation, such as delivering sensations of temperature, wetness, stickiness, magnetism (i.e., force feedback), could also help provide new avenues to enhance immersion in an entertainment context. For instance, the Ouija board experience, reported by Blenkinsopp (2019), demonstrates how tactile feedback can create a compelling sense of movement and control even without visual or auditory correlation (cf. Nielsen, 1963, for an early low-tech example of some of the unusual sensations that may be elicited when we experience a feeling of a loss of control of our own movements).

Building on the potential of diverse tactile stimuli to create engaging and immersive experiences, further research should also explore the role of touch in interactive systems, building on findings that tangible interactions can influence user affect and engagement (Spence, 2022a; Zhou et al., 2024). Obrist et al. (2016) have mentioned the importance of integrating touch with other sensory modalities, such as taste and smell, to create more meaningful multisensory experiences in HCI. By continuing to research these research avenues, we can develop more effective and immersive tactile experiences across various active entertainment mediums, ultimately enhancing user immersion, engagement, and overall experience.

## Notes

1. Though, of course, if a character were to pick up a bag of freshly-ground coffee and smell the aroma, then such product-related scent would have a particular character perspective associated with it too.

2. Note that in the field of experimental psychology, haptics typically refers to active touch (when a person moves to experience touch), while in Human-Computer Interaction (HCI), it broadly refers to any kind of tactile feedback. In this paper, we use the HCI definition of haptics.

3. Parisi (2013) proposes a game-like dimension of this setup, explaining each of the ludic elements: In this two-player game, the brave man represents one player, while the woman and the electrician together represent the other player. If the man successfully kisses the woman on the lips, he wins; if he fails to kiss her and instead receives the electric charge across the gap, they win. The latter situation represents “her ability to repel the suitor,” indicating both “her capacity to store” the “electrical fire” and the “painful passion she could unleash,” enabled by the male electrician. The insulated platform added suspense, the generation of electricity provided the game's rules and mechanics, and the kiss served as the goal and point of interaction.

4. This might even have been inspired by the 1974 film Earthquake, which is about an earthquake in California, and was one of the few films to use the innovative sound system Sensurround technique to provide the audience with vibratory sensations designed to mimic being in an earthquake (Spence & Gao, 2024b).

5. Unfortunately, however, this work never appeared in a peer-reviewed open-access format, and it is no longer accessible online, therefore making it difficult for other researchers to access and build on.

6. An observation suggest that although newer types of force feedback (such as those in the DualSense controller) are generally perceived as more immersive, many players tend to disable this feature in multiplayer games due to the additional effort required and slower response times. Instead, they typically enable the force feedback in single-player games. This hightlights how context of gameplay influences player preferences for haptic feedback.

## References

[bibr1-20416695251336117] Andrews S. Mora J. Lang J. Lee W. S. (2006). Hapticast: A physically-based 3D game with haptic feedback. Proceedings of FuturePlay 2006. University of Western Ontario.

[bibr2-20416695251336117] Bailey R. L. Kwon K. Wang T. G. Park S. Y. (2024). Eating point-of-view in ASMR videos alters motivational outcomes. Journal of Media Psychology, 36, 1–14. 10.1027/1864-1105/a000443

[bibr3-20416695251336117] Banes S. (2001). Olfactory performances. TDR/The Drama Review, 45, 68–76. 10.4324/9780203124291-52

[bibr4-20416695251336117] Birnbaum D. (2021). Haptics and gaming: A future story. *Developments in Haptics - Immersion News*. https://www.immersion.com/haptics-and-gaming-a-future-story/#:∼:text=Beyond%20airflow%20and%20temperature%2C%20new,transmitted%20in%20the%20virtual%20world.

[bibr5-20416695251336117] Blenkinsopp R. (2019, March 22). Creating haptic feedback in VR – The technology behind Ultrahaptics. *AIXR Insights*. https://aixr.org/insights/creating-haptic-feedback-in-vr-the-technology-behind-ultrahaptics/.

[bibr6-20416695251336117] Bound K. (2017). How to maximize audience engagement consistently in movies. Stage, 32.

[bibr7-20416695251336117] Buchanan L. (2012, June 15). Happy birthday, Rumble Pak. *IGN*. https://www.ign.com/articles/2008/04/03/happy-birthday-rumble-pak.

[bibr8-20416695251336117] Cashbox (1976, December 4). Sega races with ‘Fonz’ game. Cashbox, 38, 41.

[bibr9-20416695251336117] Cholewiak R. W. (2019). Do you feel… like I do? Individual differences and military multi-modal displays. In Advances in cognitive engineering and neuroergonomics (pp. 41). CRC Press.

[bibr10-20416695251336117] Costa N. (1988). Automatic pleasures: The history of the coin machine. Bath Press.

[bibr11-20416695251336117] De Menezes E. C. Lamar M. V. (2023). Haptic system as an accessibility mechanic for hard of hearing and deaf people on video games. Anais Estendidos do XXII Simpósio Brasileiro de Jogos e Entretenimento Digital (pp. 276–281). SBC.

[bibr12-20416695251336117] Desnoyers-Stewart J. Bergamo Meneghini M. Stepanova E. R. Riecke B. E. (2024). Real human touch: Performer-facilitated touch enhances presence and embodiment in immersive performance. Frontiers in Virtual Reality, 4, Article 1336581. 10.3389/frvir.2023.1336581

[bibr13-20416695251336117] Doucet N. (2024, July 29). First look: Astro Bot limited edition DualSense wireless controller*.* PlayStation Blog. Retrieved from https://blog.playstation.com/2024/07/29/first-look-astro-bot-limited-edition-dualsense-wireless-controller/.

[bibr14-20416695251336117] Earthshaker (1989, February 21). *Internet Pinball Database*. https://www.ipdb.org/machine.cgi?id=753.

[bibr15-20416695251336117] Ekman I. (2007). Sound-based gaming for sighted audiences–experiences from a mobile multiplayer location aware game. Proceedings of the 2nd Audio Mostly Conference (pp. 148–153).

[bibr16-20416695251336117] Ferrari R. (2015). Writing narrative style literature reviews. Medical Writing, 24, 230–235. 10.1179/2047480615Z.000000000329

[bibr17-20416695251336117] Furley P. Goldschmied N. (2021). Systematic vs. Narrative reviews in sport and exercise psychology: Is either approach superior to the other? Frontiers in Psychology, 12, Article 685082. 10.3389/fpsyg.2021.685082 PMC8299000 34305741

[bibr18-20416695251336117] Gallace A. Spence C. (2014). In touch with the future: The sense of touch from cognitive neuroscience to virtual reality. Oxford University Press.

[bibr19-20416695251336117] Gao Y. Spence C. (2025). Enhancing presence, immersion, and interaction in multisensory experiences through touch and haptic feedback. Virtual Worlds, 4, Article 3. 10.3390/virtualworlds4010003

[bibr20-20416695251336117] Gunther E. O’Modhrain S. (2002). Cutaneous grooves: Composing for the sense of touch. Journal of New Music Research, 32, 369–381. 10.1076/jnmr.32.4.369.18856

[bibr21-20416695251336117] Han P. H. Chen Y. S. Lee K. C. Wang H. C. Hsieh C. E. Hsiao J. C. Hung Y. P. (2018). Haptic around: Multiple tactile sensations for immersive environment and interaction in virtual reality. Proceedings of the 24th ACM Symposium on Virtual Reality Software and Technology (pp. 1–10). 10.1145/3281505.3281507

[bibr22-20416695251336117] Heilbron J. L. (1999). Electricity in the 17th and 18th centuries: A study of early modern physics. Dover Publications.

[bibr23-20416695251336117] Heilig M. (1962). *Sensorama stimulator* (U.S. Patent No. 3,050,870).

[bibr24-20416695251336117] Ho C. Spence C. (2008). The multisensory driver: Implications for ergonomic car interface design. Ashgate Publishing.

[bibr25-20416695251336117] Immersion Corporation. (2010). *Best practices for use of vibration feedback* in *console games* [White paper]. http://www.immersion.com/resources/haptic-whitepapers/#tab=rumble-best-practices.

[bibr26-20416695251336117] Ipsos-Insight (2006, September 22). *Current and next generation game console feature study. Ipsos-Insight*. https://www.ipsos.com/sites/default/files/news_and_polls/2006-09/mr060925-1report.pdf.

[bibr27-20416695251336117] Jeong S. Yun H. H. Lee Y. Han Y. (2023). Glow the buzz: A VR puzzle adventure game mainly played through haptic feedback. Extended Abstracts of the 2023 CHI Conference on Human Factors in Computing Systems (pp. 1–6).

[bibr28-20416695251336117] King D. Auschaitrakul S. You Y. (2024). Felt something, hence it works: Merely adding a sensory signal to a product improves objective measures of product efficacy and product evaluations. Journal of the Academy of Marketing Science, 52, 1761–1779. 10.1007/s11747-024-01030-z

[bibr29-20416695251336117] Kirginas S. (2022). Exploring players’ perceptions of the haptic feedback in haptic digital games. Journal of Digital Media & Interaction, 5, 7–22. 10.34624/jdmi.v5i13.30771

[bibr30-20416695251336117] Lee J. H. (2011). The expansibility of user interfaces using peripheral multisensory stimulation. HCI International 2011–Posters’ Extended Abstracts: International Conference, HCI International 2011, Orlando, FL, July 9-14, 2011, Proceedings, Part II (pp. 180–183). Berlin: Springer.

[bibr31-20416695251336117] Licata M. (2021). *Tactility, communication, connection* (Doctoral dissertation). Pratt Institute.

[bibr32-20416695251336117] Lipkin N. (2013). Controller controls: Haptics, ergon, teloi and the production of affect in the video game text. In Wysocki M. (Ed.), Ctrl-Alt-Play: Essays on control in video gaming (pp. 34–45). McFarland.

[bibr33-20416695251336117] Lovreglio R. Gonzalez V. Feng Z. Amor R. Spearpoint M. Thomas J. Trotter M. Sacks R. (2018). Prototyping virtual reality serious games for building earthquake preparedness: The Auckland City Hospital case study. Advanced Engineering Informatics, 38, 670–682. 10.1016/j.aei.2018.08.018

[bibr34-20416695251336117] Mäkelä V. Winter J. Schwab J. Koch M. Alt F. (2021). Pandemic displays: Considering hygiene on public touchscreens in the post-pandemic era. International Conference on Human Factors in Computing Systems. 10.1145/3491102.3501937.

[bibr35-20416695251336117] McAllister G. (2022, August 26^th^). *The Last of Us Part I: Full list of accessibility features.* PlayStation Blog. Sony Interactive Entertainment. Retrieved from: https://blog.playstation.com/2022/08/26/the-last-of-us-part-i-full-list-of-accessibility-features/.

[bibr36-20416695251336117] Murphy S. Dalton P. (2016). Out of touch? Visual load induces inattentional numbness. Journal of Experimental Psychology: Human Perception and Performance, 42, 761–765. 10.1037/xhp0000218 26974412 PMC4873046

[bibr37-20416695251336117] Ngo M. K. Pierce R. S. Spence C. (2012). Utilizing multisensory cues to facilitate air traffic management. Human Factors: The Journal of the Human Factors and Ergonomics Society, 54, 1093–1103. 10.1177/0018720812446 23397817

[bibr38-20416695251336117] Nielsen T. I. (1963). Volition: A new experimental approach. Scandinavian Journal of Psychology, 4, 225–230. 10.1111/j.1467-9450.1963.tb01326.x

[bibr39-20416695251336117] Obrist M. Velasco C. Vi C. T. Ranasinghe N. Israr A. Cheok A. D. Spence C. (2016). Touch, taste, & smell user interfaces: The future of multisensory HCI. Proceedings of the 2016 CHI Conference on Human Factors in Computing Systems (pp. 3285–3292). ACM. 10.1145/2851581.2856462

[bibr40-20416695251336117] Orozco M. Silva J. El Saddik A. Petriu E. (2012). The role of haptics in games. In El Saddik A. (Ed.), Haptics rendering and applications (pp. 217–234). IntechOpen. 10.5772/32809

[bibr41-20416695251336117] Palan S . (2020). *Tactile gaming vest (TGV)*. iRoboticist. https://iroboticist.com/tactile-gaming-vest-tgv/.

[bibr42-20416695251336117] Paris U. Milan U. Reflections U. Montreal U. Bucharest U. Montpellier U. Pune U. Barcelona U. Shanghai U. Bordeaux U. (2009–2025). Just Dance [Video game]. Ubisoft.

[bibr500-20416695251336117] Paris D. (2009). Gameinterfaces as bodily techniques. In R. E. Ferdig (Ed.), Handbook of research on effective electronic gaming in education (Vol. 1, pp. 111–126). IGI Global.

[bibr43-20416695251336117] Parisi D. (2013). Shocking grasps: An archaeology of electrotactile game mechanics. Game Studies, 13, Retrieved from http://gamestudies.org/1302/articles/parisi.

[bibr44-20416695251336117] Parisi D. (2015). A counterrevolution in the hands: The console controller as an ergonomic branding mechanism. Journal of Games Criticism, 2, 1–23.

[bibr45-20416695251336117] Parisi D. (2018). Archaeologies of touch: Interfacing with haptics from electricity to computing. University of Minnesota Press.

[bibr46-20416695251336117] Parisi D. (2019). Rumble/control: Toward a critical history of touch feedback in video games. ROMchip, 1.

[bibr47-20416695251336117] PatmanQC (2019). *The history of the Fonz arcade racer - documentary* [Video]. YouTube. https://www.youtube.com/watch?v=KvDZmVFrdXM.

[bibr48-20416695251336117] Pescara E. Wolpert A. Budde M. Schankin A. Beigl M. (2017). Lifetact: Utilizing smartwatches as tactile heartbeat displays in video games. Proceedings of the 16th International Conference on Mobile and Ubiquitous Multimedia (pp. 97–101).

[bibr49-20416695251336117] Pinball Cabinet (2023). Haptic feedback in virtual pinball: Bridging the gap to realism. *Pinball Cabinet Blog*. https://pinballcabinet.com/blogs/news/haptic-feedback-in-virtual-pinball-bridging-the-gap-to-realism.

[bibr50-20416695251336117] Pozo T. (2018). Queer games after empathy: Feminism and haptic game design aesthetics from consent to cuteness to the radically soft. Game Studies, 18, Retrieved from https://gamestudies.org/1803/articles/pozo/.

[bibr51-20416695251336117] Pritchett D. Gallace A. Spence C. (2011). Implicit processing of tactile information: Evidence from the tactile change detection paradigm. Consciousness and Cognition, 20, 534–546. 10.1016/j.concog.2011.02.006 21459622

[bibr52-20416695251336117] Pursey T. Lomas D. (2018). Tate sensorium: An experiment in multisensory immersive design. The Senses and Society, 13, 354–366. 10.1080/17458927.2018.1516026

[bibr53-20416695251336117] Seedhouse A. (2017, January 15^th^). Immersion’s TouchSense technology used in Nintendo Switch. Nintendo Insider.

[bibr54-20416695251336117] Söderström U. Larsson W. Lundqvist M. Norberg O. Andersson M. Mejtoft T. (2022). Haptic feedback in first person shooter video games. Proceedings of the 33rd European Conference on Cognitive Ergonomics (pp. 1–6).

[bibr55-20416695251336117] Sony Interactive Entertainment Europe (2023). Horizon Call of the Mountain. PlayStation. Retrieved from https://www.playstation.com/en-gb/games/horizon-call-of-the-mountain/.

[bibr56-20416695251336117] Sony Interactive Entertainment (n.d.). DualSense^TM^ wireless controller. PlayStation. Retrieved from https://www.playstation.com/en-gb/accessories/dualsense-wireless-controller/.

[bibr57-20416695251336117] Spence C. (2014). The skin as a medium for sensory substitution. Multisensory Research, 27, 293–312. 10.1163/22134808-00002452 25693298

[bibr58-20416695251336117] Spence C. (2020). Scent and the cinema. i-Perception, 11, 1–22. 10.1177/20416695209697 PMC7691926 33282170

[bibr59-20416695251336117] Spence C. (2021). Scent in the context of live performance. i-Perception, 12, 1–28. 10.1177/2041669520985537 PMC7871084 33613954

[bibr60-20416695251336117] Spence C. (2022a). Multisensory contributions to affective touch. Current Opinion in Behavioral Sciences, 43, 40–45. 10.1016/j.cobeha.2021.08.003

[bibr61-20416695251336117] Spence C. (2022b). Proprioceptive art: How should it be defined, and why has it become so popular? i-Perception, 13, 1–22. 10.1177/20416695221120522 PMC9459486 36092512

[bibr62-20416695251336117] Spence C. (2022c). From the fairground sensorium to the digitalization of bodily entertainment: Commercializing multisensory entertainments involving the bodily senses. The Senses and Society, 17, 153–169. 10.1080/17458927.2022.2066434

[bibr63-20416695251336117] Spence C. Gallace A. (2007). Recent developments in the study of tactile attention. Canadian Journal of Experimental Psychology / Revue Canadienne de Psychologie Expérimentale, 61, 196–207. 10.1037/cjep2007021 17974314

[bibr64-20416695251336117] Spence C. Gao Y. (2024a). Enhancing public entertainment with touch: Possibilities and pitfalls. i-Perception, 15, 1–25. 10.1177/20416695241280715 PMC11489974 39431169

[bibr65-20416695251336117] Spence C. Gao Y. (2024b). Augmenting home entertainment with digitally-delivered touch. i-Perception, 15, 1–27. 10.1177/20416695241281474 PMC11490966 39431170

[bibr66-20416695251336117] Spence C. Ho C. (2008). Crossmodal information processing in driving. In Castro C. (Ed.), Human factors of visual performance in driving (pp. 187–200). CRC Press.

[bibr67-20416695251336117] Spence C. Ngo M. K. (2012). Does attention or multisensory integration explain the cross-modal facilitation of masked visual target identification? In Stein B. E. (Ed.), The new handbook of multisensory processing (pp. 345–358). MIT Press.

[bibr68-20416695251336117] Takeshita Y. Miyake H. (2020). Animal Crossing: New Horizons [Video game]. Nintendo.

[bibr69-20416695251336117] Tao X. Wu K. Yang Y. (2021). The effects of vibration on assisting game play and improving player engagement when lacking sound. In International conference on human-computer interaction (pp. 389–407). Springer International Publishing.

[bibr70-20416695251336117] TNT Amusements Inc (2013). #287 Williams EARTHSHAKER Pinball Machine - first machine with SHAKER Motor! [Video]. YouTube. https://www.youtube.com/watch?v=4LxmdEMBY_0 .

[bibr71-20416695251336117] TX-1. *Wikipedia*. https://en.wikipedia.org/wiki/TX-1.

[bibr72-20416695251336117] Van der Burg E. Olivers C. N. L. Bronkhorst A. W. Theeuwes J. (2009). Poke and pop: Tactile-visual synchrony increases visual saliency. Neuroscience Letters, 450, 60–64. 10.1016/j.neulet.2008.11.002 19013216

[bibr74-20416695251336117] Weinstein S. (1968). Intensive and extensive aspects of tactile sensitivity as a function of body part, sex, and laterality. In Kenshalo D. R. (Ed.), The skin senses (pp. 195–222). Thomas.

[bibr75-20416695251336117] Willumsen E. C. Jaćević M. (2019). A typology of rumble. Proceedings of DiGRA 2019.

[bibr76-20416695251336117] Wilska A. (1954). On the vibrational sensitivity in different regions of the body surface. Acta Physiologica Scandinavica, 31, 285–289. 10.1111/j.1748-1716.1954.tb01139.x 13197098

[bibr77-20416695251336117] Woojer (n.d.). *Vest: Feel sound like never before*. Retrieved from https://www.woojer.com/products/vest.

[bibr78-20416695251336117] Zhang Y. Wang Y. Liu Q. (2022). Touch the history in virtuality: Combine passive haptic with 360 videos in history learning. 2022 IEEE Conference on Virtual Reality and 3D User Interfaces Abstracts and Workshops (VRW) (pp. 824–825). IEEE. 10.1109/VRW55335.2022.00263

[bibr79-20416695251336117] Zhou N. Devleminck S. Geurts L. (2024, July). Tangible affect: A literature review of tangible interactive systems addressing human core affect, emotions, and moods. Proceedings of the 2024 ACM Designing Interactive Systems Conference (pp. 424–440).

